# Efficacy of Tranexamic Acid in Reducing Blood Loss During Percutaneous Nephrolithotomy: An Interventional Study

**DOI:** 10.7759/cureus.85863

**Published:** 2025-06-12

**Authors:** Meenal Meharwal, Rajeev Sarpal, Shikhar Agarwal, Meghali Dhebane, Jitendra Prasad Ray, Rishin Dutta, Divyanshu Joshi

**Affiliations:** 1 General Surgery, Swami Rama Himalayan University, Dehradun, IND; 2 Urology, Swami Rama Himalayan University, Dehradun, IND; 3 Pathology, Swami Rama Himalayan University, Dehradun, IND

**Keywords:** blood loss, efficacy, percutaneous nephrolithotomy (pcnl), safety, tranexamic acid (txa)

## Abstract

Background: An interventional study was conducted in the Department of Urology at a tertiary care center in Dehradun, India, to assess the efficacy of tranexamic acid (TXA) in reducing blood loss during percutaneous nephrolithotomy (PCNL).

Materials and methods: A total of 99 patients with renal calculi were recruited for this study over a period of 18 months. Subjects were selected from patients presenting with a history of flank pain in the Outpatient Department (OPD). Written informed consent for the inclusion of patient data in the study was obtained from all participants. The study was undertaken after obtaining ethical clearance from the Institutional Ethics Committee.

Results: In this study, there was no statistical difference in gender between the groups receiving TXA and those not receiving it (p-value = 0.492). The mean age of the recruited cases in both groups was between 42 and 43 years. This study recorded calyceal puncture sites, irrigating fluid used, and pre- and postoperative hemoglobin and packed cell volume (PCV) values to calculate blood loss. Additionally, body mass index (BMI), co-morbidities, stone size, ease of puncture and dilatation, and operative time were recorded as other variables for both groups. There was no mortality observed in any of the patients postoperatively. Blood loss was much lower in patients receiving TXA.

Conclusion: TXA is safe and effective in reducing blood loss during PCNL.

## Introduction

Urinary stone disease is very common worldwide. Literature shows that in India, approximately two million people are affected by urolithiasis each year [1]. After infections and prostate disease, urinary stones rank as the third most prevalent disease of the urinary tract [2].

To achieve complete stone clearance, it is crucial to be free from any pathogens, prevent additional stone formation, address any related infections, and maintain kidney function. Treatment options for stone clearance include percutaneous nephrolithotomy (PCNL), extracorporeal shock wave lithotripsy (ESWL), antegrade ureteroscopy (URS), and retrograde intrarenal surgery (RIRS). According to international guidelines, the standard of care for treating stones larger than 2 cm in the upper urinary tract is PCNL because it has a higher stone clearance rate. In our study, stone clearance was defined as no stone or clinically insignificant fragments (<4 mm) visible on non-contrast computed tomography (NCCT) of the kidneys, ureters, and bladder (KUB) during follow-up at one month. A good PCNL is characterized by obtaining secure percutaneous renal access. A common method for entering the pelvicalyceal system is through fluoroscopic or ultrasound (USG) guidance, which can be performed by either urologists or radiologists. A new prognostic method for forecasting PCNL outcomes is staghorn morphometry [3].

Rationale and knowledge gap

Hemorrhage is the most serious complication of PCNL. Both patients and surgeons experience stress when bleeding occurs following PCNL [4].

During PCNL, bleeding can happen due to initial puncture, percutaneous tract dilatation, and injury to vessels caused by manipulating the nephroscope during surgery. An increase in urine fibrinolytic activity is hypothesized to be linked to postoperative blood loss in urological procedures. High levels of plasminogen activators found in urine and urothelium make clot lysis easier [5].

Some methods to prevent perioperative blood loss include using allogenic blood products to replace lost blood, employing hypotensive anesthesia, and using antifibrinolytic drugs like tranexamic acid (TXA), epsilon aminocaproic acid (EACA), and aprotinin after surgery. Ultimately, prevention with a cost-effective and safe medication like TXA could prove to be the cornerstone for developing future guidelines. TXA is a "non-competitive inhibitor of plasmin at larger doses, while it is a competitive inhibitor of plasminogen activation at lower concentrations” [4].

According to a systematic analysis of randomized clinical studies, TXA reduced the likelihood of needing a blood transfusion by one-third in patients undergoing elective surgery [6]. Researchers have previously explored hemostatic drugs intended to reduce tract-site bleeding. The rate of perirenal hematoma was not significantly decreased with the local use of TachoSil in tubeless PCNL [7]. The administration of TXA may influence postoperative gross hematuria, one of the major factors affecting hospital stay duration. This could explain the relationship between TXA usage and a shorter length of hospital stay. Patients receiving TXA during various urological procedures have not been found to have dissolvable blood clots present, which could impair the surgeon's vision. Preventing this complication with a reliable and affordable medication like TXA could prove to be a critical component for developing new PCNL recommendations [8].

Coagulopathy, acute lung injury, a hemolysis response, non-immune hemolysis, infections, and mistransfusion are among the dangers associated with blood transfusions that impose a significant financial burden on society. For TXA to be effective, it must first bind to plasminogen. This binding prevents plasmin from interacting with fibrin, thereby inhibiting the breakdown of fibrin clots. Minimizing perioperative blood loss is one of the objectives during the recovery phase post-surgery.

TXA has been frequently utilized in hip and knee replacement surgeries. It has a multimodal mechanism of action and is a unique treatment for melasma. The primary use of oral TXA is in the acute treatment of excessive menstrual bleeding. TXA is widely used and readily available, and it appears on the World Health Organization (WHO)'s list of essential medications. Improved bleeding management could potentially reduce trauma-related mortality by 10-20%, according to a TXA study (CRASH-2 trial) [9]. TXA has shown promise in managing hemorrhage in adult trauma patients. In patients with renal calculi receiving PCNL, we aimed to investigate the effect of TXA on hemoglobin levels and bleeding. All surgeons involved in this study had more than 10 years of experience performing PCNL. This study was conducted to determine the effectiveness of TXA in minimizing blood loss during PCNL and the need for blood transfusions, if any, following this procedure.

## Materials and methods

Objectives

The study aimed to assess the effectiveness of TXA in lowering the amount of blood lost during PCNL.

Methods

The study was observational in nature. The administration of TXA was determined entirely by the operating surgeons as part of their routine clinical practice at the Himalayan Institute of Medical Sciences, Swami Rama Himalayan University in Dehradun, India. The use of TXA is standard practice at the institution and was not influenced or directed by the study investigators. The operating surgeons, who were responsible for patient management and intraoperative decision-making, were not involved in the study design, data collection, or analysis. Accordingly, the allocation of the intervention was not controlled by the authors, ensuring that the study remained observational in its methodology.

The sample size for this study was 99 (53 patients in group A and 46 in group B). Inclusion criteria for the study included patients undergoing elective PCNL for symptomatic urolithiasis and aged 18 years and older. Exclusion criteria encompassed individuals with a serum creatinine level greater than 1.5 mg/dL, patients with congenital renal anomalies, those with bleeding disorders, sepsis, undergoing anticoagulation therapy, and any individuals with contraindications to the use of TXA.

The study was conducted over a duration of 18 months and received approval in November 2021.

Study protocol

Institutional Ethics Committee approval was obtained prior to conducting the study. Patients were recruited from the Outpatient Department (OPD) or the indoor facility of the Department of Urology. Diagnostic modalities used included ultrasound, X-ray KUB, and CT urography/intravenous pyelography (IVP). CT Hounsfield units, stone complexity, and calyceal anatomy were reported in detail by the reporting radiologist, which helped the operating surgeon to plan their puncture techniques and reduce puncture technique-related bleeding, thereby minimizing bias.

Patients were treated by the treating surgeon with treatment as usual. Demographic data, stone clearance (no stone or fragments <4 mm on NCCT KUB at one month), disease-related variables, treatment history, and family history of medical disorders were recorded.

All eligible patients undergoing PCNL, selected at the discretion of the operating surgeon, were prospectively observed over a one-month period. Patients were categorized into two groups based on whether they received an intravenous dose of TXA (1 g in 10 mL) administered 20 minutes prior to the surgical procedure (group A) or not (group B). Patients were closely monitored for side effects like tachycardia, anxiety, confusion, etc.

General anesthesia or spinal anesthesia was administered according to the patient’s preference and their medical condition. A single dose of cefuroxime 1 g was given as prophylaxis during the perioperative period. Blood samples were collected preoperatively, postoperatively, and 24 hours postoperation. Preoperative hemoglobin level, irrigating fluid volume, and total fluid volume used during the surgery were documented. The hemoglobin measurement was performed by a pathologist in the Department of Pathology using standard approved machines. From the above measurements, blood loss was calculated as follows:

\[ \text{Calculated blood loss (L)} = \frac{\text{Hb concentration in irrigating fluid (mg dL)} \times \text{irrigating fluid volume (L)}}{\text{Hb in blood at preoperation (g dL)} \times {10^3}} \]

**Figure 1:**
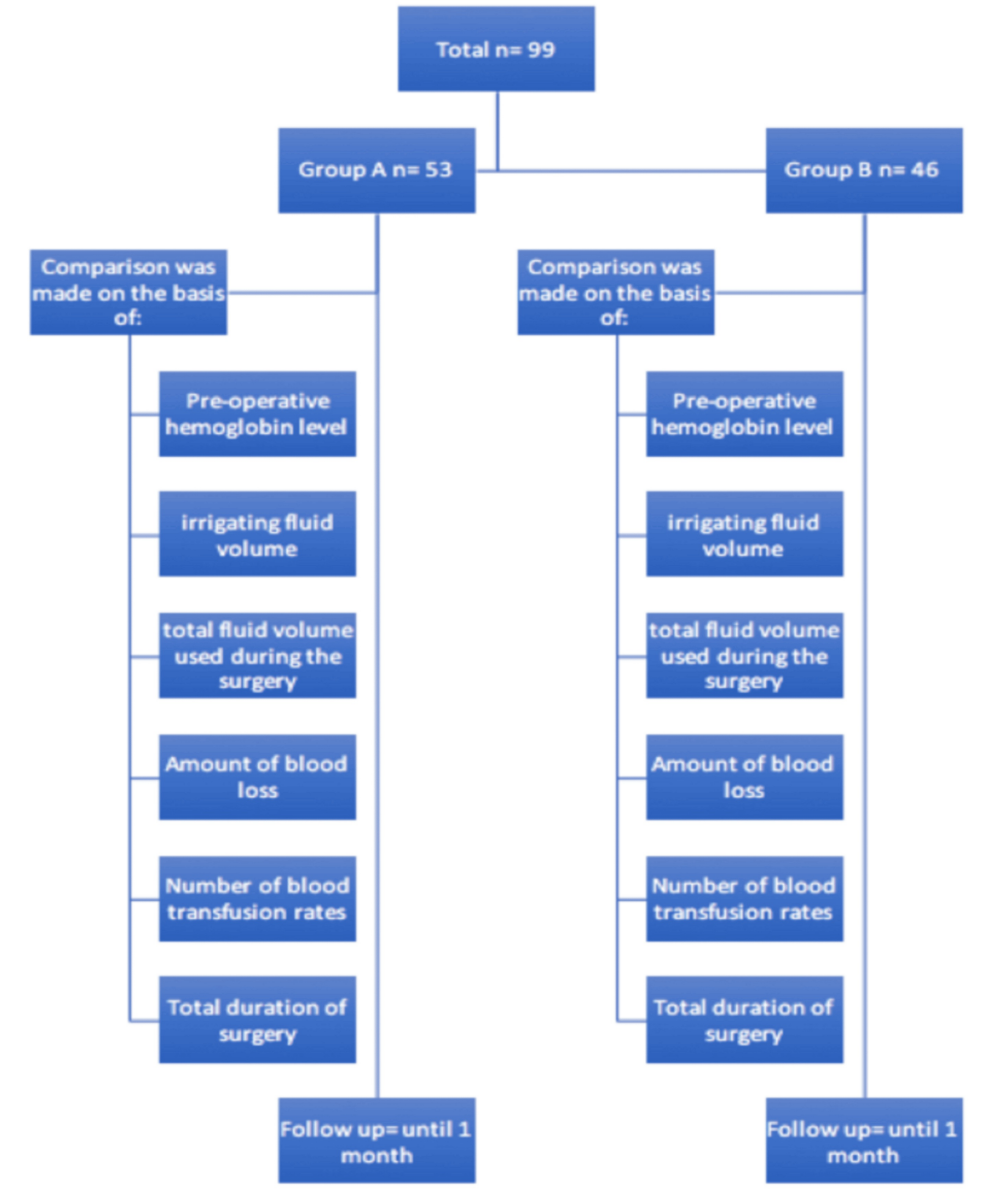
CONSORT diagram CONSORT: Consolidated Standards of Reporting Trials

The total duration of surgery was noted. To analyze postoperative complications, the modified Clavien-Dindo classification system was used. All patients who underwent PCNL were followed up until one month after the operative procedure (Figure 1). The amount of blood loss during surgery was measured by assessing hemoglobin levels, which were entered in the chart, and statistical analysis was conducted.

Data analysis

IBM SPSS Statistics version 22 (IBM Corp., Armonk, USA) was used for analysis. Statistical significance was defined as a p-value less than 0.05.

## Results

Subject distribution

The sample size for this study was 99 patients, with 53 in group A and 46 in group B.

Distribution of Subjects as per Gender

As shown in Table 1, group A comprised 58.49% (n=31) male patients, while group B had 65.22% (n=30) male patients.

**Table 1:** Distribution of subjects as per gender (n=99) Data represented as n (%). A p-value of <0.05 was considered significant.

Parameters	Group A	Group B
Male	31 (58.49%)	30 (65.22%)
Female	22 (41.51%)	16 (34.78%)
Total	53 (53.53%)	46 (46.46%)
p-value	0.492	

Distribution of Subjects as per Age Group

**Figure 2:**
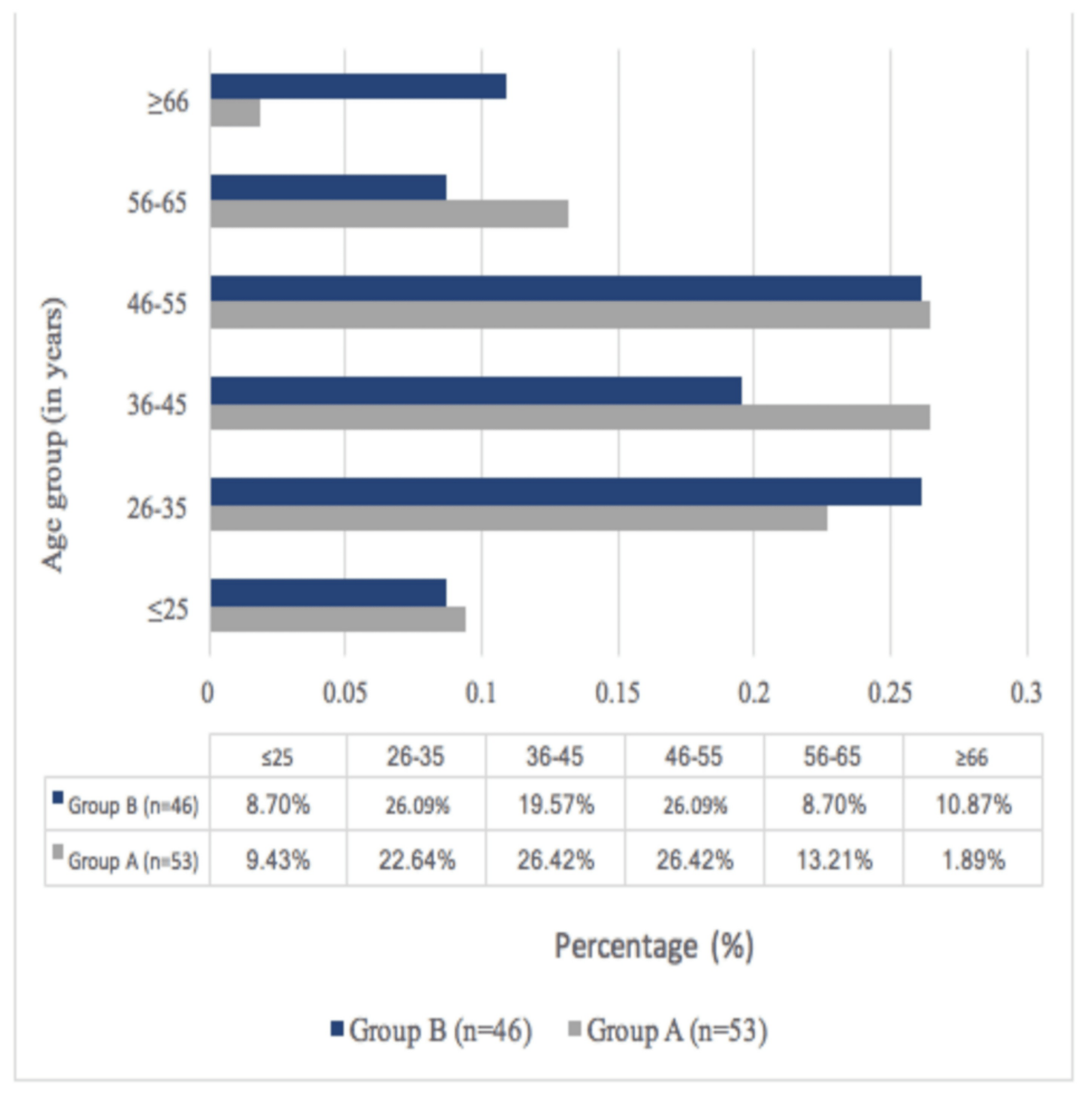
Distribution of subjects as per age group

Of the 99 patients included in this study, the majority, 26.42% (n=26), were observed in the age group of 46-55 years in group A and 26.09% in group B (Figure 2).

Distribution of Subjects as per Body Mass Index (BMI)

There was no significant difference in BMI between the groups, with p-values of 0.25 (Table 2).

**Table 2:** Distribution of subjects as per BMI group Data represented as mean ± SD. A p-value of <0.05 was considered significant. BMI: Body mass index

Parameters	Group A	Group B	p-value
BMI (in kg/m^2^)	23.72 ± 0.97	23.6 ± 0.88	0.25

Medical history of study participants

**Figure 3:**
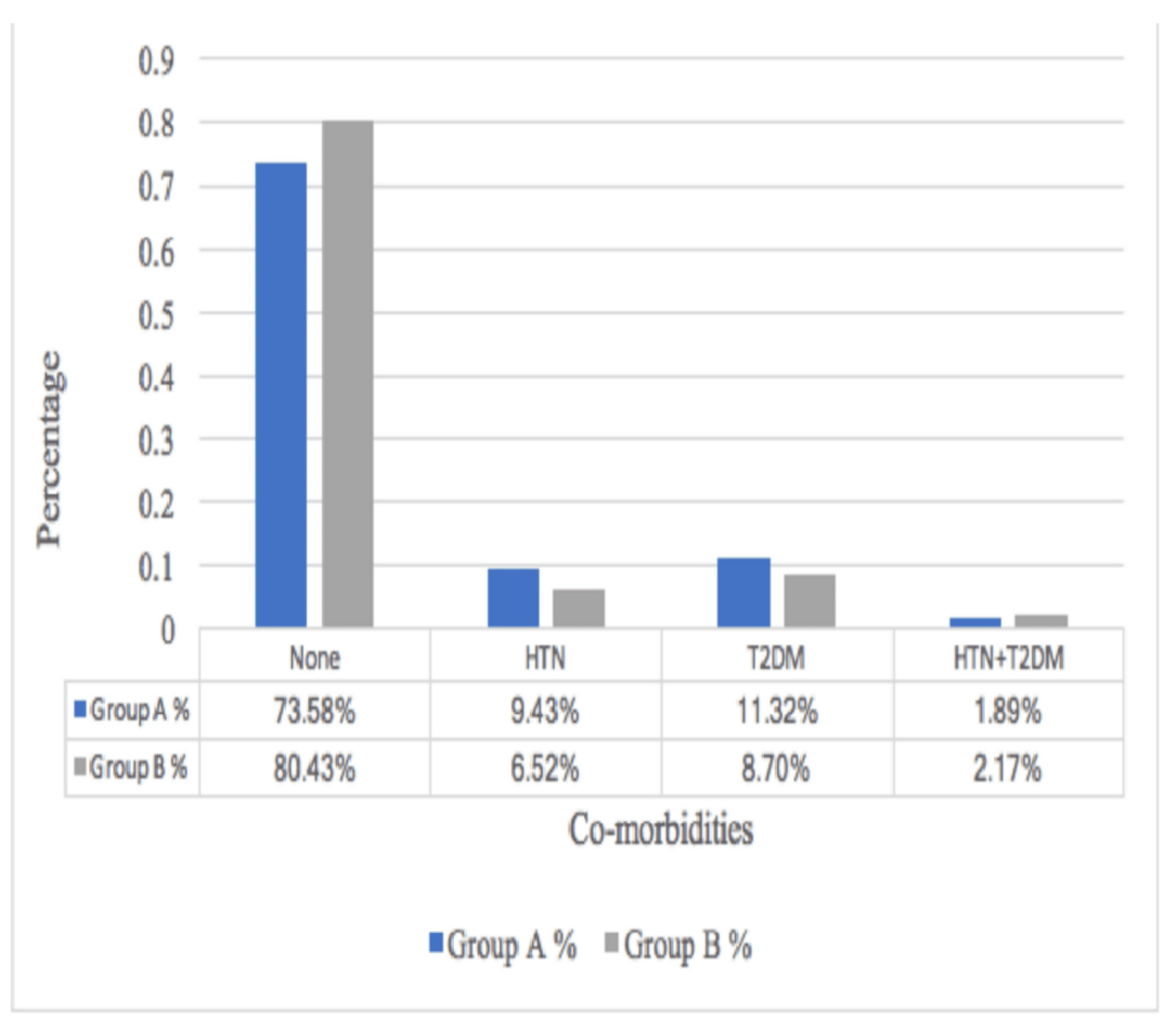
Medical history of study participants HTN: Hypertension; T2DM: Type 2 diabetes mellitus

As shown in Figure 3, most of the study participants had no associated co-morbidities, with 73.58% (n=39) in group A and 80.43% (n=37) in group B.

Distribution of Subjects According to Symptoms

**Figure 4:**
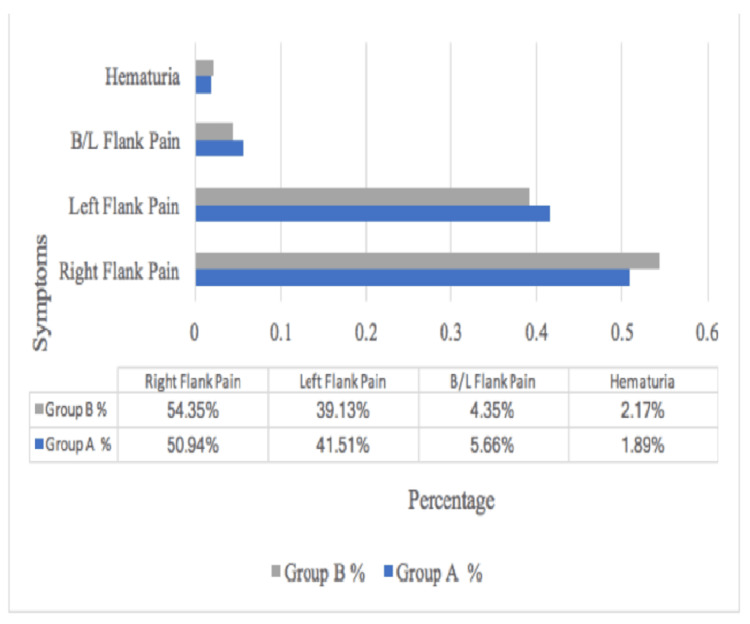
Distribution of subjects according to symptoms B/L: Bilateral

Right flank pain was the most common symptom among the study subjects, with group A showing 50.94% compared to group B at 54.35%. Group A reported 41.51% left flank pain compared to 39.13% in group B. Furthermore, 5.66% of group A participants experienced bilateral flank pain, which was higher than the 4.35% observed in group B. Only 2% of patients in each group presented with hematuria (Figure 4).

Distribution of Subjects According to Operative Parameters Related to Number and Site of Puncture

The majority of cases involved a single puncture, with 88 patients (88.9%) experiencing this, while 11 patients (11.1%) had two punctures. The lower calyx was the primary site of puncture for 45.65% of group B subjects, compared to 33.96% of group A subjects. Punctures in the middle calyx were performed in 19 cases (19.2%), multiple calyces in 12 cases (12.1%), and the upper calyx in five cases (5.1%) (Table 3).

**Table 3:** Distribution of subjects according to operative parameters in relation to number and site of puncture A p-value of <0.05 was considered significant.

Variable		Group A (n=53)		Group B (n=46)		p-value
		n	%	n	%	
No. of puncture	1	47	88.68%	41	89.13%	0.943
	2	6	11.32%	5	10.87%	
Site of puncture	Lower calyx	18	33.96%	21	45.65%	0.122
	Middle calyx	16	30.19%	14	30.43%	
	Upper calyx	14	26.42%	4	8.70%	
	Multiple calyces	5	9.43%	7	15.22%	

Distribution of Subjects According to Amplatz Size

**Figure 5:**
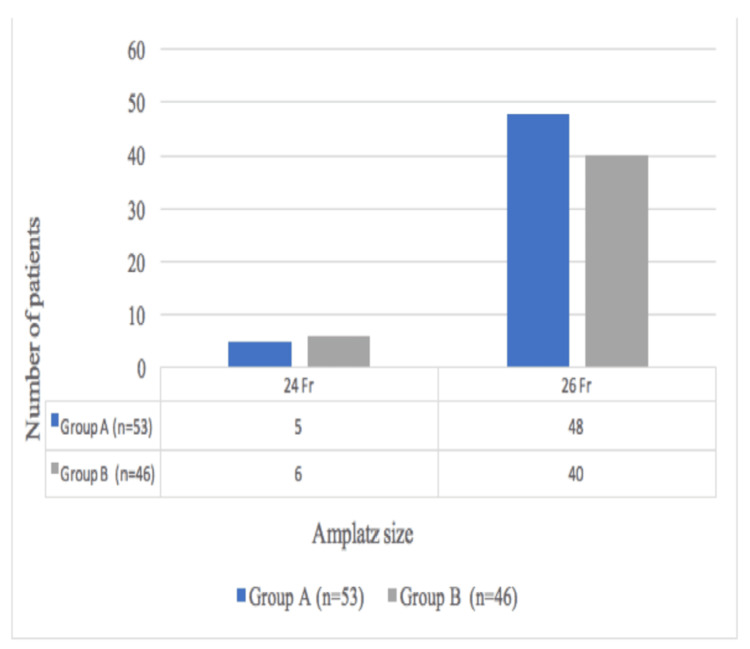
Distribution of subjects according to Amplatz size

There was no significant difference in Amplatz size between the two groups (p=0.568) (Figure 5).

Distribution of Subjects According to Average Stone Size

Group A had an average stone size of 2.19 ± 0.91 cm, while group B had an average stone size of 2.30 ± 1.10 cm (Table 4).

**Table 4:** Distribution of subjects according to stone size Data represented as mean ± SD. A p-value of <0.05 was considered significant.

Parameters	Group A	Group B	p-value
Stone size (in cm)	2.19 ± 0.91	2.30 ± 1.10	0.45

Subject Distribution According to Irrigating Fluid Amount 

The amount of irrigating fluid used was 11.89 ± 1.67 L in group A and 13.62 ± 1.68 L in group B, which was statistically significant (p=0.001) (Table 5).

**Table 5:** Distribution of subjects according to amount of irrigating fluid used Data represented as mean ± SD. A p-value of <0.05 was considered significant.

Parameters	Group A	Group B	p-value
Amount of irrigating fluid (L)	11.89 ± 1.67	13.62 ± 1.68	0.001

Comparison Between Laboratory and Operative Parameters of Patients Receiving TXA vs. Placebo

The preoperative hemoglobin (g/dL) and preoperative packed cell volume (PCV) showed no significant difference between both groups (p=0.286, 0.355). However, postoperative hemoglobin (g/dL), postoperative PCV, and the amount of blood lost were significantly different between group A and group B (p=0.038, 0.045, 0.001, respectively). The average blood loss was observed to be lower in group A (Table 6).

**Table 6:** Comparison between laboratory and operative parameters of patients receiving TXA vs. placebo Data represented as mean ± SD. A p-value of <0.05 was considered significant. TXA: Tranexamic acid; PCV: Packed cell volume; Hb: Hemoglobin

Parameters	Group A	Group B	p-value
Preoperative Hb (g/dL)	12.97 ± 2.00	12.53 ± 2.11	0.286
Preoperative PCV (%)	39.49 ± 5.74	38.37 ± 6.21	0.355
Postoperative Hb (g/dL)	12.56 ± 1.99	11.72 ± 2.15	0.038
Postoperative PCV (%)	37.67 ± 5.81	35.53 ± 6.04	0.045
Estimated blood loss (L)	155 ± 68	252 ± 103	0.001

Comparison Between Laboratory and Operative Parameters With Respect to Fall in Hemoglobin and PCV

The fall in hemoglobin (g/dL) and the fall in PCV between both groups showed a statistically significant difference (p=0.001) (Table 7).

**Table 7:** Comparison between laboratory and operative parameters of patients receiving TXA vs. placebo with respect to fall in Hb and PCV Data represented as mean ± SD. A p-value of <0.05 was considered significant. TXA: Tranexamic acid; PCV: Packed cell volume; Hb: Hemoglobin

Parameters	Group A	Group B	p-value
Fall in Hb (g/dl)	0.40 ± 0.24	0.81 ± 0.56	0.001
Fall in PCV (%)	1.81 ± 3.10	2.84 ± 1.66	0.001

Comparison Between Laboratory and Operative Parameters With Respect to Site of Puncture

There was a statistically significant difference in postoperative hemoglobin (p=0.004 and 0.008) and postoperative PCV (p=0.022 and 0.012), irrespective of which calyx was punctured. The estimated blood loss was significantly different between group A and group B (p=0.005) (Table 8).

**Table 8:** Comparison between laboratory and operative parameters of patients receiving TXA vs. placebo with respect site of puncture Data represented as mean ± SD. A p-value of <0.05 was considered significant. TXA: Tranexamic acid; PCV: Packed cell volume; Hb: Hemoglobin

Parameters		Group A	Group B	p-value
Preoperative Hb (g/dL)	Lower calyx	12.94 ± 2.04	12.45 ± 2.05	0.389
	Middle calyx	13.90 ± 1.80	12.45 ± 1.97	0.045
	Upper calyx	13.93 ± 1.68	11.61 ± 1.22	0.022
Preoperative PCV (%)	Lower calyx	39.54 ± 5.76	38.24 ± 6.09	0.424
	Middle calyx	42.23 ± 4.89	38.06 ± 5.73	0.041
	Upper calyx	42.53 ± 4.63	36.18 ± 3.88	0.024
Postoperative Hb (g/dL)	Lower calyx	12.48 ± 2.04	11.59 ± 1.24	0.041
	Middle calyx	13.41 ± 1.85	11.16 ± 2.09	0.004
	Upper calyx	13.47 ± 1.75	10.70 ± 0.91	0.008
Postoperative PCV (%)	Lower calyx	38.08 ± 5.76	35.21 ± 4.85	0.036
	Middle calyx	39.22 ± 6.26	33.86 ± 5.83	0.022
	Upper calyx	40.76 ± 5.16	32.90 ± 3.40	0.012
Estimated blood loss (L)	Lower calyx	145 ± 62	249 ± 133	0.001
	Middle calyx	140 ± 54	244 ± 113	0.002
	Upper calyx	144 ± 54	246 ± 103	0.016

Comparison Between Patients Receiving TXA vs. Placebo With Respect to Operative Time

A statistically significant difference in mean operating time was observed between the two groups (Table 9).

**Table 9:** Comparison between patients receiving TXA vs. placebo with respect to operative time A p-value of <0.05 was considered significant. TXA: Tranexamic acid; PCV: Packed cell volume; Hb: Hemoglobin

Parameter	Group A	Group B	p-value
	Mean ± SD	Mean ± SD	
Operative time (in min)	53.66 ± 18.796	58.74 ± 20.110	0.0197

Comparison Between Patients Receiving TXA vs. Placebo With Respect to Stone Clearance

**Figure 6:**
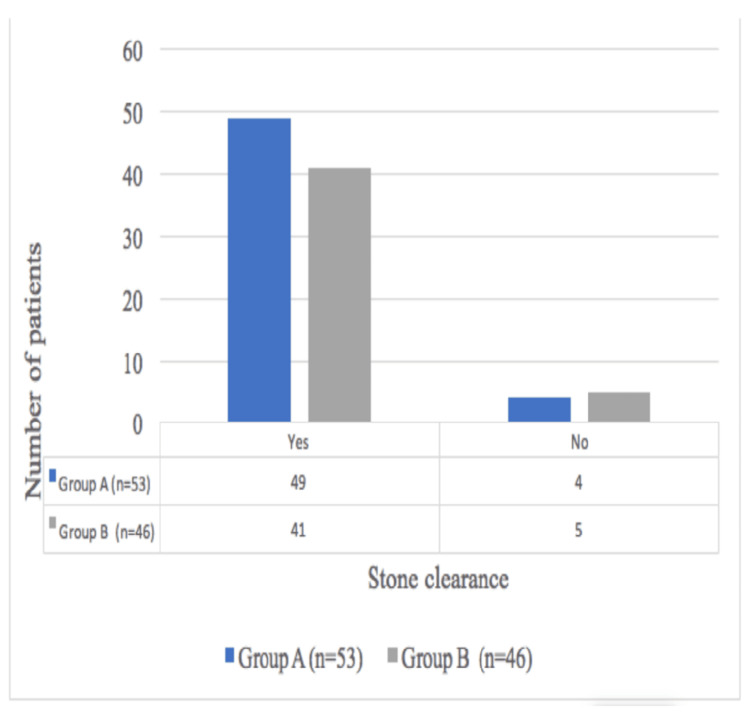
Comparison between patients receiving TXA vs. placebo with respect to stone clearance TXA: Tranexamic acid

Stone clearance showed no significant difference between the two groups (p=0.470) (Figure 6). In group A, four patients had incomplete clearance, of which three were managed conservatively and one underwent staged PCNL. In group B, five patients had incomplete clearance, with three managed conservatively, one underwent staged PCNL, and one underwent ESWL.

## Discussion

PCNL is the preferred treatment for large upper tract urolithiasis. Although this process has advanced greatly due to technical improvements, several complications still occur [10]. In PCNL, damage occurs to the interlobular and segmental vessels upon entry into the collecting system, during tract dilatation, and intrarenal maneuvers like nephroscopy, leading to significant bleeding. Hemorrhage is the most frequent complication, and when conservative measures fail, it often necessitates blood transfusion and angiographic embolization [11].

Bansal and Arora [12] used Amplatz fascial dilators for tract dilatation; however, in our study, we employed telescopic Alken dilators for the same purpose. Securing the best possible approach to the collecting system is essential for reducing bleeding and achieving a successful PCNL; surgical knowledge is a probable element that may influence the risk of hemorrhage [12].

The motivation for conducting this study stemmed from specific findings in the literature. During our research, only one international study published by Kumar et al. [13] was identified. Some critical surgical parameters, such as calculi site and volume, puncture techniques, dilator size and type, and Amplatz sheath size, were insufficiently addressed in the Kumar et al. [13] study. We believe these factors are vital since they serve as important confounders and have an impact on the study's findings.

One of the most significant side effects of PCNL is bleeding, which can usually be managed with simple conservative treatment or blood transfusions. Blood loss management is crucial to PCNL. Bleeding is commonly observed during the puncture of the collecting system, tract dilatation, nephroscopy, and disintegration of calculus. Various studies have reported a transfusion rate ranging from 3% to 23% [14,15]. Kukreja investigated blood loss during PCNL and identified factors associated with it. The author noted prolonged operative time, multiple tracts, and the frequency of intraoperative complications as contributing factors [16].

However, in rare cases, open procedures or angiographic intervention may be necessary [11]. For patients with genetic bleeding disorders and women experiencing excessive menstrual bleeding, TXA was initially administered [17]. The European Association of Urology (EAU) guidelines indicate that ongoing anticoagulant therapy, untreated urinary tract infections (UTIs), tumors in the presumed access tract area, possible malignant kidney tumors, and pregnancy serve as contraindications for PCNL treatment [18].

Most bleeding that occurs during PCNL can be managed with appropriate medical interventions, such as inserting a larger nephrostomy tube, clamping the nephrostomy tube, and administering fluids, mannitol, and Kaye balloon tamponade. Venous bleeding is often the cause and can typically be managed using these techniques [18].

Kumar et al. [13] were the first to report TXA as a safe and useful medication for reducing blood loss associated with PCNL. They also found that patients receiving TXA experienced a lesser drop in hemoglobin level (1.39 g/dL) compared to the placebo group (2.31 g/dL; p<0.0001). They stated that the drug is well tolerated and leads to lower blood loss and fewer complications. In our study, patients receiving TXA showed a drop in hemoglobin of 0.40 ± 0.24 g/dL and a decrease in PCV of 1.81 ± 3.10 (p=0.001).

Benefits of TXA during PCNL were similarly reported by Akman et al. [19]. The TXA group exhibited a significantly lower hemoglobin drop and reduced transfusion rate in comparison to the control group. Siddiq et al. [20] also demonstrated that administering TXA prior to surgery can significantly reduce hemoglobin drop (median 1.6 g/dL vs. 1.3 g/dL, p=0.001) and transfusion rate (75% vs. 25%, p=0.038). In the current study, patients who received TXA had an average estimated blood loss of 155 ± 68 mg/dL. The difference in blood loss was statistically significant between the two groups. Postoperative hemoglobin (gm/dL), postoperative PCV, and estimated blood loss showed statistically significant differences between both groups (p=0.038, 0.045, 0.001). Overall mean scores were higher in group A for all parameters except for estimated blood loss, which was lower in group A.

In a separate study, Lee et al. found that despite training, only 27% of urologists continued to perform percutaneous access during residency. Inadequate skills (33%) or the radiologist's superior abilities (44%) were two of the explanations offered in their study [21]. In our study, preoperative hemoglobin exhibited a significant difference for the middle calyx and upper calyx (0.041, 0.022), while preoperative PCV also showed a significant difference for these areas (0.041, 0.024). Postoperative hemoglobin demonstrated significant differences across all calyces (0.004, 0.008), and postoperative PCV showed significant contrasts for all calyces (0.022, 0.012). Estimated blood loss revealed significant variations with respect to all puncture sites (p=0.005). The mean operative time in our study showed a decreasing trend in group A (53.66 ± 18.79 vs. 58.74 ± 20.11 minutes, p=0.197), unlike Kumar et al. [13], where the mean operative time was significantly reduced. This finding may be explained by the fact that administering TXA improves the vision of the operating surgeon.

We conducted a comparison between patients receiving TXA vs. placebo regarding stone clearance, and no significant difference was noted between the two groups (p=0.470). In group A, four patients had incomplete clearance, three of whom were managed conservatively, and one underwent staged PCNL. In group B, five patients experienced incomplete clearance, with three managed conservatively, one underwent staged PCNL, and one was treated with ESWL.

A recent randomized controlled study from North India by Bansal and Arora reported that TXA reduced the rates of blood transfusions and blood loss. The authors also noted reduced operative time and early hospital discharge. However, in this trial, TXA was administered intravenously rather than through irrigation fluid, which resulted in a drop in hemoglobin levels and decreased blood loss in the TXA group [12].

Strengths of the study

Our study provides an impactful clinical outcome with comprehensive data collection. Quantitative measurements of blood loss were conducted both preoperatively and post-procedure, which helped determine the efficacy of TXA in PCNL.

Limitations of the study

This is a single-center study with a limited sample size. Moreover, only short-term outcomes were evaluated; due to the brief follow-up period, long-term outcomes were not assessed.

## Conclusions

For renal stones larger than 2 cm, PCNL is the procedure of choice. Bleeding is the most common complication of PCNL. Bleeding during surgery may hamper the operative vision and lead to incomplete stone clearance, subsequently resulting in the need for staged PCNL. This bleeding causes a drop in hemoglobin levels, which may sometimes necessitate blood transfusions during the postoperative period. A safe and effective method for reducing blood loss during PCNL is TXA, and the estimated blood loss showed a significant difference between the two groups in our study (p=0.005).
